# Supplementary material for: Magic spreading in random quantum circuits
Source: Nat Commun. 2025 Mar 15;16:2575. doi: 10.1038/s41467-025-57704-x (PMC11910662; doi:10.1038/s41467-025-57704-x)
Supplement: Supplementary file 1 — Supplementary Information [file 41467_2025_57704_MOESM1_ESM.pdf]

Supplementary Information for “Magic spreading in random quantum circuits”

Xhek Turkeshi,[1,*] Emanuele Tirrito,[2,3] and Piotr Sierant[4,5,†]

[1] Institut für Theoretische Physik, Universität zu Köln, Zülpicher Strasse 77, 50937 Köln, Germany
[2] The Abdus Salam International Centre for Theoretical Physics (ICTP), Strada Costiera 11, 34151 Trieste, Italy
[3] Pitaevskii BEC Center, CNR-INO and Dipartimento di Fisica, Università di Trento, Via Sommarive 14, Trento, I-38123, Italy
[4] ICFO-Institut de Ciències Fotòniques, The Barcelona Institute of Science and Technology, Av. Carl Friedrich Gauss 3, 08860 Castelldefels, Barcelona, Spain
[5] Barcelona Supercomputing Center Plaça Eusebi Güell, 1-3 08034, Barcelona, Spain

SUPPLEMENTARY NOTE 1: SELECTED PROPERTIES OF THE CLIFFORD GROUP AND ITS COMMUTANTS

The Clifford group, a subgroup of unitary operations $\mathcal{C}_{N,d} \subset \mathcal{U}(d^N)$, maps a Pauli string to another single Pauli string, up to a phase $CPC^\dagger = \omega^r P$, with $r \in \mathbb{Z}_d$. It is completely generated by the Hadamard, CADD, and phase gates, respectively

$$\begin{aligned} H &= \frac{1}{\sqrt{d}} \sum_{m,n=0}^{d-1} \omega^{mn} |m\rangle\langle n| \,, \quad \mathrm{CADD} = \sum_{m,n=0}^{d-1} |m, m \oplus_d n\rangle\langle m,n| \,, \\ P &= |0\rangle\langle 0| + i|1\rangle\langle 1| \;\text{ for } d=2, \qquad P = \sum_{m=0}^{d-1} \omega^{m(m-1)2^{-1}} |m\rangle\langle m| \;\text{ otherwise,} \end{aligned} \tag{1}$$

where 2^{-1} represents the multiplicative inverse in the finite field $\mathbb{Z}_d$. With a slight abuse of notation, we will move between these notations, inferring the specific representation from the context. We define for later convenience $D = 2d$ for $d = 2$ and $D = d$ for $d \geq 3$.

The generalized stabilizer entropies (GSEs) are intimately connected to the commutant of the Clifford group, meaning those operators W acting on $(\mathcal{H}_d^{\otimes N})^{\otimes k}$ such that $[W, C^{\otimes k}] = 0$ for any $C \in \mathcal{C}_{N,d}$. We briefly review the key properties and results on the Clifford commutant, cf. Ref. [1, 2] for an indepth analysis. The Schur-Weyl duality links the k-commutant to the set $\Sigma_k(d)$ of stochastic Lagrangian subspaces of $\mathbb{Z}_d^{2k}$, namely the subspaces $T \subset \mathbb{Z}_d^{2k}$ such that: (i) any $(\mathbf{x},\mathbf{y}) \in T$ has $\mathbf{x}\cdot\mathbf{x} - \mathbf{y}\cdot\mathbf{y} = 0 \bmod D$, T has dimension $\dim T = k$ in the field $\mathbb{Z}_d^{2k}$ and $\mathbf{1}_{2k} \equiv (1,1,\dots,1) \in T$. These spaces induce operators $w(T) = \sum_{(\mathbf{x},\mathbf{y})\in T} |\mathbf{x}\rangle\langle\mathbf{y}|$ acting on $\mathcal{H}_d^{\otimes k}$, with $|\mathbf{x}\rangle = |x_1,\dots,x_k\rangle$ a vector associated with $\mathbf{x} \in \mathbb{Z}_d^k$. Similarly, we can construct an N-qudit operator in the k-replica space $W(T) = w(T)^{\otimes N}$. Both $w(T)$ and $W(T)$ are real matrices in the computational basis $\{|\mathbf{x}\rangle \mid \mathbf{x} \in \mathbb{Z}_d^k\}$ and $\{|\mathbf{x}^{(1)}, \mathbf{x}^{(2)}, \cdots, \mathbf{x}^{(N)}\rangle \mid \mathbf{x}^{(j)} \in \mathbb{Z}_d^k\}$, respectively.

To demonstrate that the operators $W(T) \in \mathrm{Comm}_k(\mathcal{C}_{N,d})$ for any $T \in \Sigma_k(d)$, it is sufficient to show that $[G, W(T)] = 0$ for the generators $G \in \{H, P, \mathrm{CADD}\}$, which span the whole Clifford group. Consider the orthogonal space $T^\perp = \{(x,y) \in \mathbb{Z}_d^{2k} \mid \mathbf{x}\cdot\mathbf{x}' = \mathbf{y}\cdot\mathbf{y}' \bmod d \text{ for any } (\mathbf{x},\mathbf{y}) \in T\}$. From the definition of stochastic Lagrangian subspaces, it follows that

$$(\mathbf{x}+\mathbf{x}')\cdot(\mathbf{x}+\mathbf{x}') - (\mathbf{y}+\mathbf{y}')\cdot(\mathbf{y}+\mathbf{y}') = 0 \bmod D = 2(\mathbf{x}\cdot\mathbf{x}' - \mathbf{y}\cdot\mathbf{y}') \bmod d = \mathbf{x}\cdot\mathbf{x}' - \mathbf{y}\cdot\mathbf{y}' \bmod d \,, \tag{2}$$

implying $T \subset T^\perp$, i.e., T is self-orthogonal. Furthermore, since $\dim T + \dim T^\perp = 2k$ and $\dim T = k$, it follows that $\dim T^\perp = k$, meaning that $T = T^\perp$. This remark allows us to show that, for the Hadamard gate

$$H^{\otimes k} w(T) (H^\dagger)^{\otimes k} = \frac{1}{d^k} \sum_{\mathbf{m},\mathbf{n}\in\mathbb{Z}_d} \sum_{(\mathbf{x},\mathbf{y})\in T} \omega^{\mathbf{m}\cdot\mathbf{x} - \mathbf{n}\cdot\mathbf{y}} |\mathbf{m}\rangle\langle\mathbf{n}| = \sum_{(\mathbf{x},\mathbf{y})\in T^\perp} |\mathbf{x}\rangle\langle\mathbf{y}| = w(T). \tag{3}$$

For the phase gate, simple algebraic manipulations in the qubit case where $d = 2$

$$P^{\otimes k} w(T) (P^\dagger)^{\otimes k} = \sum_{(\mathbf{x},\mathbf{y})\in T} i^{\mathbf{x}\cdot\mathbf{x} - \mathbf{y}\cdot\mathbf{y}} |\mathbf{x}\rangle\langle\mathbf{y}| = \sum_{(\mathbf{x},\mathbf{y})\in T} |\mathbf{x}\rangle\langle\mathbf{y}| = w(T), \tag{4}$$

* turkeshi@thp.uni-koeln.de
† piotr.sierant@bsc.es

while for $d \geq 3$, a prime number, we have instead

$$P^{\otimes k} w(T)(P^{\dagger})^{\otimes k} = \sum_{(\mathbf{x},\mathbf{y})\in T} \omega^{2^{-1}\sum_{m=0}^{d-1}[x_m(x_m-1)-y_m(y_m-1)]} |\mathbf{x}\rangle\langle\mathbf{y}| = \sum_{(\mathbf{x},\mathbf{y})\in T} \omega^{-2^{-1}(\mathbf{x}\cdot\mathbf{1}_k - \mathbf{y}\cdot\mathbf{1}_k)} |\mathbf{x}\rangle\langle\mathbf{y}| = w(T). \tag{5}$$

Finally, for the CADD, we consider $w(T)^{\otimes 2}$ and find

$$\mathrm{CADD}^{\otimes k} w(T)^{\otimes 2} (\mathrm{CADD}^{\dagger})^{\otimes k} = \sum_{(\mathbf{x},\mathbf{y})\in T} \sum_{(\mathbf{x}',\mathbf{y}')\in T} |\mathbf{x}, \mathbf{x}+\mathbf{x}'\rangle\langle\mathbf{y}, \mathbf{y}+\mathbf{y}'| = \sum_{(\mathbf{x},\mathbf{y})\in T} \sum_{(\mathbf{z},\mathbf{w})\in T} |\mathbf{x}, \mathbf{z}\rangle\langle\mathbf{y}, \mathbf{w}| = w(T)^{\otimes 2}, \tag{6}$$

concluding the proof.

The operators $W(T)$ induced by the stochastic lagrangian subspaces completely characterize the commutant, since, for $N \geq k-1$, $|\Sigma_k(d)| = |\mathrm{Comm}_k(\mathcal{C}_{N,d})| = \prod_{i=0}^{k-2}(d^i + 1)$. As a consequence, identifying the operators in $\mathrm{Comm}_k(\mathcal{C}_{N,d})$ amounts to the characterization of $T \in \Sigma_k(d)$. We note that $\Sigma_k(d)$ is not a group, and in particular, the operators $W(T)$ are not generally invertible. We define the stochastic orthogonal group $\mathcal{O}_k$ as the set of $k \times k$ matrices with entries in the field $\mathbb{Z}$ such that $O\mathbf{x} \cdot O\mathbf{x} - \mathbf{x} \cdot \mathbf{x} = 0 \bmod D$ for all $\mathbf{x} \in \mathbb{Z}_d^k$ and $O\mathbf{1}_k = \mathbf{1}_k \bmod d$. Any operator $O \in \mathcal{O}_k$ has its graph $T_O \equiv \{(O\mathbf{x}, \mathbf{x}) \mid \mathbf{x} \in \mathbb{Z}_d^k\} \in \Sigma_k(d)$ and $w(O) \equiv w(T_O)$ is invertible and orthogonal. From the group $\mathcal{O}_k$, we can define the left and right group actions $OT = \{(O\mathbf{x}, \mathbf{y}) \mid (\mathbf{x}, \mathbf{y}) \in T\}$ and $TO = \{(\mathbf{x}, O^T\mathbf{y}) \mid (\mathbf{x}, \mathbf{y}) \in T\}$, both elements in $\Sigma_k(d)$. This action is consistent with the composition of the operators $W(T)$ and $W(O)$, namely $W(O)W(T)W(O') = W(OTO')$ for any $O, O' \in \mathcal{O}_k$ and $T \in \Sigma_k(d)$. Therefore, the left and right actions allow for the decomposition of $\Sigma_k(d)$ into a disjoint union of double cosets

$$\Sigma_k(d) = \mathcal{O}_k \sqcup \mathcal{O}_k T_1 \mathcal{O}_k \sqcup \cdots \sqcup \mathcal{O}_k T_r \mathcal{O}_k \tag{7}$$

for some r, and $T_1, \ldots, T_r \in \Sigma_k(d)$ as choices of representatives in different cosets, and $\mathcal{O}_k$ is regarded as a subset of $\Sigma_k(d)$ under the identification $O \mapsto T_O$. A relevant subgroup of $\mathcal{O}_k$ that is also stochastic and orthogonal are the permutation operators $\pi \in S_k \subset \mathcal{O}_k$, playing a fundamental role in the Schur-Weyl duality for the Haar group. En passant, we note that the intrinsic Clifford commutant contains all operators $W(T)$ induced by $T \in \Sigma_k(d) \setminus S_k$.

Remarkably, the number of subspaces T_r that generate disjoing double cosets is bounded to $r \leq k$ [1]. To see this, we recall that all elements of $\Sigma_k(d)$ can be expressed in terms of defect subspaces. Specifically, for any $T \in \Sigma_k(d)$, we define its left and right defect subspaces, respectively, $\mathcal{L}(T) \equiv \{\mathbf{x} \in \mathbb{Z}_d^k \mid (\mathbf{x}, \mathbf{0}) \in T\}$ and $\mathcal{R}(T) \equiv \{\mathbf{y} \in \mathbb{Z}_d^k \mid (\mathbf{0}, \mathbf{x}) \in T\}$. Furthermore, for every $\mathbf{y} \in \mathcal{R}(T)^{\perp}$, the map $\mathcal{J}[\mathbf{y}] = [\mathbf{x}(\mathbf{y})]$ such that $(\mathbf{x}(\mathbf{y}), \mathbf{y}) \in T$ is a well-defined defect isomorphism between $\mathcal{R}(T)$ and $\mathcal{L}(T)$. A stochastic Lagrangian subspace T is uniquely determined by the triplet $(\mathcal{L}(T), \mathcal{R}(T), \mathcal{J})$. One can further show that $\mathcal{J}$ can be induced by a stochastic orthogonal matrix $O \in \mathcal{O}_k$. Since orthogonal transformations preserve rank, it follows that for any $T, T' \in \Sigma_{k,k}(d)$, we have $T' = OT$ for a certain $O \in \mathcal{O}_k T$ if and only if $\dim \mathcal{L}(T) = \dim \mathcal{L}(T')$ and $\mathbf{1}_k \in \dim \mathcal{L}(T)$ if and only if $\mathbf{1}_k \in \dim \mathcal{L}(T')$. This remark implies that $\Sigma_k(d)$ can have at most k cosets, as $\dim \mathcal{L}(T) \leq k$ for any $T \in \Sigma_k(d)$. The operators $w(T)$ induced by the stochastic Lagrangian subspace T are expressible in terms of the coset states. For any defect subspace

$$|\mathcal{A}, [\mathbf{x}]\rangle = \frac{1}{\sqrt{|\mathcal{A}|}} \sum_{\mathbf{z}\in\mathcal{A}} |\mathbf{x} + \mathbf{z}\rangle \ , \tag{8}$$

which form an orthonormal family for $[\mathbf{x}] \in \mathfrak{A} = \mathcal{A}^{\perp}/\mathcal{A}$. Then for T characterized by $(\mathcal{L}(T), \mathcal{R}(T), \mathcal{J})$, we have

$$w(T) = |\mathcal{L}(T)| \sum_{[\mathbf{y}]\in\mathcal{R}(T)^{\perp}/\mathcal{R}(T)} |\mathcal{L}(T), [\mathbf{x}(\mathbf{y})]\rangle\langle\mathcal{R}(T), [\mathbf{y}]| \ . \tag{9}$$

A convenient choice of operators are Calderbank-Shor-Steane (CSS) codes, characterized by the left and right defect subspaces coinciding, i.e., $\mathcal{A} \equiv \mathcal{L}(T) = \mathcal{R}(T)$. In this case, the projector onto the code space satisfies

$$Q_{\mathcal{A}} = \frac{1}{|\mathcal{A}|^2} \sum_{\mathbf{p},\mathbf{q}\in\mathcal{A}} Z_{\mathbf{q}} X_{\mathbf{p}} = \sum_{[\mathbf{x}]\in\mathfrak{A}} |\mathcal{A}, [\mathbf{x}]\rangle\langle\mathcal{A}, [\mathbf{x}]| \ , \tag{10}$$

and, for the associated operator $w(T) = |\mathcal{A}| Q_{\mathcal{A}}$. Any such stochastic Lagrangian subspace T identifies a *unique* double coset of $\Sigma_k(d)$.

Finally, the set of subspaces $\Sigma_k(d)$ can be endowed with a semigroup structure $\circ$ such that $T \mapsto w(T)$ is a representation

$$w(T_1)w(T_2) = |\mathcal{A}_1 \cap \mathcal{A}_2| w(T_1 \circ T_2) \ . \tag{11}$$

Case 1.— When T_1 is associated with $O \mapsto T_1$, then $T_1 \circ T_2 \equiv OT$. A similar reasoning applies when T_2 is the subspace associated with an isometry. *Case 2.*— When T_1 and T_2 are associated with (coinciding left and right) defect subspaces $\mathcal{A}_1$ and $\mathcal{A}_2$ respectively, we define $T_1 \circ T_2$ as the stochastic Lagrangian subspace T fixed by the triple

$$\begin{aligned} \mathcal{L}(T) &\equiv (\mathcal{A}_1 + \mathcal{A}_2) \cap \mathcal{A}_1^{\perp}, \\ \mathcal{R}(T) &\equiv (\mathcal{A}_1 + \mathcal{A}_2) \cap \mathcal{A}_2^{\perp}, \\ \mathcal{J}&: \mathcal{R}(T)^{\perp}/\mathcal{R}(T) \to \mathcal{L}(T)^{\perp}/\mathcal{L}(T), \quad [\mathbf{y}] \mapsto [\mathbf{x}(\mathbf{y})], \end{aligned} \tag{12}$$

where $\mathbf{x}$ is such that $\mathbf{x} - \mathbf{y} \in \mathcal{A}_1 + \mathcal{A}_2$. Arbitrary elements T_1 and T_2 in $\Sigma_k(d)$ can be recast in the two cases above via an isometry $\mathcal{O}_k$. In conclusion, the Clifford commutant is completely determined by the stochastic orthogonal group $\mathcal{O}_k$ and by at most $r \leq k$ defect subspace projectors. The above ingredient provides a complete characterization of the Clifford commutant. In the Main Text, we inferred the dependence $W = W(T)$ of the stochastic lagrangian subspace T to lighten the notation. L. Leone and collaborators will present a simplified but equivalent construction is presented in a forthcoming publication.

A Identifying Defect Subspaces

While the orthogonal group $\mathcal{O}_k$ on d dimensional qudits are well known [1], the remaining terms in the Clifford commutant requires the computation of the defect subspaces corresponding to a CSS code. The brute force cost of identifying such defect subspaces grows exponentially as d^k. This process involves examining integers $n = 0, \dots, d^k - 1$, expressing each n as $\sum_{i=1}^{k} b_i d^{i-1}$ in the D-basis, resulting in a vector $\mathbf{v}^{(n)} \equiv (b_1, b_2, \dots, b_k)$. Each vector is checked to satisfy the conditions $\mathbf{v}^{(n)} \cdot \mathbf{v}^{(n)} = 0 \mod D$ and $\mathbf{v}^{(n)} \cdot \mathbf{1}_k = 0 \mod d$. We compile a list of such vectors $\mathcal{S} = \{\mathbf{v}^{(n_1)}, \mathbf{v}^{(n_2)}, \dots, \mathbf{v}^{(n_K)}\}$, reducing it to a number $\tilde{K}$ of linearly independent vectors. Each subset $\mathcal{S}_{\mathcal{A}}$, for which any $\mathbf{x}, \mathbf{y} \in \mathcal{S}_{\mathcal{A}}$ satisfies (i) $\mathbf{x} \cdot \mathbf{y} = 0 \mod d$ and (ii) $\mathbf{z} = \mathbf{x} + \mathbf{y} \mod d$ with $\mathbf{z} \cdot \mathbf{z} = 0 \mod D$ and $\mathbf{z} \cdot \mathbf{1}_k = 0 \mod d$, defines a defect subspace $\mathcal{A} = \mathrm{span}(\mathcal{S}_{\mathcal{A}})$.

To illustrate this concept, consider the case $d = 2$ and $k = 5$. By brute force evaluation, we find:

$$\tilde{\mathcal{S}} = \{(1,1,1,1,0), (1,0,1,1,1), (1,1,0,1,1), (1,1,1,0,1), (1,1,1,1,0)\}. \tag{13}$$

The only subset of mutually orthogonal vectors, representing defect subspaces, includes those with a single generator, such as:

$$\mathcal{A}_{(1,1,1,1,0)} \equiv \mathrm{span}(\{(1,1,1,1,0)\}), \quad \mathcal{A}_{(1,1,1,0,1)} \equiv \mathrm{span}(\{(1,1,1,0,1)\}), \quad \text{etc.}\,. \tag{14}$$

A noteworthy case arises for $k = 6$ replicas and $d = 2$, where:

$$\tilde{\mathcal{S}} = \{(1,1,1,1,0,0),\ +\ \text{permutations}\}, \tag{15}$$

resulting in 15 elements. Each element of $\tilde{\mathcal{S}}$ generates a one-dimensional defect subspace, e.g., $\mathcal{A}_{(1,1,1,1,0,0)} \equiv \mathrm{span}(\{(1,1,1,1,0,0)\})$. However, subsets $\mathcal{S}_{\mathcal{A}}$ with two elements induce 9 distinct 2-dimensional defect subspaces, such as:

$$\mathcal{A} = \mathrm{span}(\{(1,1,1,1,0,0), (1,1,0,0,1,1)\}). \tag{16}$$

Interestingly, $\mathbf{1}_6$ is not a defect subspace, but corresponds to the anti-identity matrix, an element of $\mathcal{O}_6$ [1]. Similarly, all transposition (swaps) correspond to vectors $(1,-1,0,0,\dots)$ and their permutations. Similarly, for $d = 3$ and $k = 4, 5$, only one-dimensional defect subspaces exist. At $k = 6$, however, we encounter a two-dimensional space generated by:

$$\mathcal{A} = \mathrm{span}(\{(1,1,1,0,0,0), (0,0,0,1,1,1)\}), \tag{17}$$

and other permutations. This notation translates to a recipe to write $W(T)$ in the Pauli basis in replica space, cf. also the forthcoming publication of L. Leone and collaborators for an in-depth discussion. Any commutant operator amounts to

$$W(T) = \sum_{P_1,\dots,P_r \in \mathcal{P}_N(d)} \frac{1}{d^{Nr}} P_1^{\otimes v_1} \otimes P_2^{\otimes v_2} \otimes \cdots \otimes P_r^{\otimes v_r} \otimes I^{\otimes k - \sum_j v_j}, \tag{18}$$

up to a permutation operators $W(T_\pi)$ with $\pi \in S_k \subset \Sigma_k(d)$. Here, the integers $v_j \in \mathbb{Z}_d$, determined by the stochastic Lagrangian subspace T, are positive $v_j > 0$, and satisfy $\sum_j v_j \leq k$ and $\sum_j v_j = 0 \mod d$.

SUPPLEMENTARY NOTE 2: GENERALIZED STABILIZER ENTROPIES AS GOOD MEASURES OF MAGIC

We consider a pure state $|\Psi\rangle$ and recall that, for any intrinsic Clifford commutant operator W the definition of the GSEs and associated generalized stabilizer purities

$$M_W(|\Psi\rangle) = -\log[\zeta_W(|\Psi\rangle)], \quad \zeta_W(|\Psi\rangle) = \mathrm{Tr}(W|\Psi\rangle\langle\Psi|^{\otimes k}). \tag{19}$$

The GSEs posses the following properties as nonstabilizerness measures.
Clifford invariance.— Since $W \in \overline{\mathrm{Comm}_k}(\mathcal{C}_{N,d}) \subset \mathrm{Comm}_k(\mathcal{C}_{N,d})$, for any $C \in \mathcal{C}_{N,d}$ a Clifford unitary

$$\zeta_W(C|\Psi\rangle) = \mathrm{tr}(W(C|\Psi\rangle\langle\Psi|C^\dagger)^{\otimes k}) = \mathrm{tr}\left[(C^\dagger)^{\otimes k} W C^{\otimes k}(|\Psi\rangle\langle\Psi|)^{\otimes k}\right] = \mathrm{tr}(W(|\Psi\rangle\langle\Psi|)^{\otimes k}) = \zeta_W(|\Psi\rangle)\ .$$

Hence ζ_W and, thus, M_W are invariant under Clifford conjugation.
Additivity.— Consider two states $|\psi\rangle$ and $|\phi\rangle$ defined respectively in $\mathcal{H}_d^{\otimes N_A}$ and $\mathcal{H}_d^{\otimes N_B}$ with $N = N_A + N_B$. Since $W = w^{\otimes N}$ is a tensor product over qudits, we have

$$\begin{aligned} M_W(|\psi\rangle \otimes |\phi\rangle) &= -\log\left[w^{\otimes N}\left[(|\psi\rangle \otimes |\phi\rangle)(\psi| \otimes \langle\phi|)\right]^{\otimes k}\right] \\ &= -\log\left[w^{\otimes N_A}(|\psi\rangle\langle\psi|)^{\otimes k}\right] - \log\left[w^{\otimes N_B}(|\phi\rangle\langle\phi|)^{\otimes k}\right] = M_W(|\psi\rangle) + M_W(|\phi\rangle) \end{aligned}$$

where we used basic properties of the logarithm function.
Faithfulness.— We note that $|\Psi\rangle \in \mathrm{STAB}_{N,d}$ iff $M_W(|\Psi\rangle) = 0$ for $W \in \overline{\mathrm{Comm}}_k(\mathcal{C}_{N,d})$ is equivalent to $|\Psi\rangle \in \mathrm{STAB}_{N,d}$ iff $\zeta_W(|\Psi\rangle) = 1$ for $W \in \overline{\mathrm{Comm}}_k(\mathcal{C}_{N,d})$. Suppose that $|\Psi\rangle \in \mathrm{STAB}_{N,d}$ is a stabilizer state. This implies existence of a Clifford transformation such that $|0\rangle^{\otimes N} = C|\Psi\rangle$. Being W in the commutant for a certain $T \in \Sigma_k(d)$, we have

$$\zeta_W = \mathrm{tr}(W(T)|\Psi\rangle\langle\Psi|^{\otimes k}) = \langle 0|^{\otimes Nk} W(T)|0\rangle^{\otimes Nk} = \langle \mathbf{0}_k|w(T)|\mathbf{0}_k\rangle^{\otimes N} = 1\ , \tag{20}$$

where in the last step we used that $\mathbf{0}_k \in T$ for any $T \in \Sigma_k(d)$. Vice-versa, consider $\zeta_W(|\Psi\rangle) = 1$ for $W \in \overline{\mathrm{Comm}}_k(\mathcal{C}_{N,d})$. (For $W \in \mathrm{Comm}_k(\mathcal{U}(d^N))$ this is identically true for any state.) Using permutation invariance $1 = \zeta_W = \zeta_{\tilde{W}}$ with $\tilde{W}$ in the form Eq. (18). Thus

$$1 = \zeta_W(|\Psi\rangle) = \mathrm{Tr}[\tilde{W}|\Psi\rangle\langle\Psi|^{\otimes k}] = \frac{1}{d^{Nr}} \sum_{P_1,\ldots,P_r} \prod_{j=1}^{r} \beta_j^{v_j}\ , \tag{21}$$

where we used $|\Psi\rangle\langle\Psi| = \sum_P \beta_P P/d^N$ with $\beta_P = \langle\Psi|P^\dagger|\Psi\rangle$. Furthermore, being a pure state, $\sum_P |\beta_P|^2 = d^N$. This constraint, together with the absolute value of Eq. (21) implies that $|\beta_P| = 1$ for d^N terms and zero otherwise. To fix the phases, we note that $|\beta_P| = 0, 1$ in Eq. (21) induces an infinite hierarchy of constraints $1 = \zeta_{W^{(q)}}(|\Psi\rangle)$ when considering $k \mapsto qk$ replicas, with

$$W^{(q)}(T) = \sum_{P_1,\ldots,P_r \in \mathcal{P}_N(d)} \frac{1}{d^{Nr}} P_1^{\otimes q v_1} \otimes P_2^{\otimes q v_2} \otimes \cdots \otimes P_r^{\otimes q v_r} \otimes I^{\otimes qk - \sum_j (q v_j)}, \tag{22}$$

and all possible permutation in S_{qk}. Recalling $P^d = I$, this hierarchy holds if and only if $\beta_P = \omega^{m_P}$ for some $m_P \in \mathbb{Z}_d$. Thus, $|\Psi\rangle\langle\Psi| = \sum_P \omega^{m_P} P/d^N$, implying $|\Psi\rangle \in \mathrm{STAB}_{N,d}$, as required.

SUPPLEMENTARY NOTE 3: MONOTONES OF MAGIC STATE RESOURCE THEORY

In the following, we analyze the properties of GSEs from the point of view of magic state resource theory. A real-valued function $f(\rho)$ is a stabilizer monotone if, for any state ρ and a stabilizer protocol $\mathcal{E} : \rho \to \mathcal{E}(\rho)$

$$f(\mathcal{E}(\rho)) \leq f(\rho). \tag{23}$$

Our manuscript focuses on the spreading of magic resources under many-body dynamics. Hence, we are mostly interested in the protocols $f(.)$ corresponding to unitary evolution. Nevertheless, to put our considerations into a broader context, we investigate the properties of GSEs under the general stabilizer protocols. Recently, we remark that, for qubits, SREs have been proved as monotone [3] when involving also the computational basis measurements. Here we generalize the proof to d dimensional qudits, and reveal with concrete counterexamples that GSEs are not generally monotones.

A Stabilizer Rényi entropies are magic monotone for qudits

In this section, we prove that the stabilizer Rényi entropy is a magic monotone for any qudit dimension d prime. The idea is based on Ref. [3], and required bounding the expectation value $P_\alpha(\Psi) \equiv d^{-N}\sum_{P\in\mathcal{P}_N}|\langle\Psi|P|\Psi\rangle|^{2\alpha}$ for a state on N qudits $|\Psi\rangle = \sum_{i=0}^{d-1}\sqrt{p_i}|i\rangle\otimes|\phi_i\rangle$ with $\sum_{i=0}^{d-1}p_i = 1$, $\{|i\rangle\}_{i=0,\dots,d-1}$ on-site state in the computational basis, and $\{|\phi_i\rangle\}_{i=0,\dots,d-1}$ generic states on the remaining $N-1$ qudits. In particular, we have that the stabilizer Rényi entropy is a magic monotone if the following Lemma holds:

Lemma 1. *Consider* $|\Psi\rangle = \sum_{i=0}^{d-1}\sqrt{p_i}|i\rangle\otimes|\phi_i\rangle$ *and* $|\phi_i\rangle\in\mathbb{C}^{d\otimes(N-1)}$*. For any integer* $\alpha\geq 2$ *it holds that*

$$P_\alpha(\Psi)\leq\max_j\{P_\alpha(\phi_j)\}\,. \tag{24}$$

Proof. Expanding $P_\alpha(\Psi)$ over $\tilde{P}\otimes P$ with $P\in\mathcal{P}_{N-1}$ and $\tilde{P}\in\mathcal{P}_1$ we have

$$P_\alpha(\Psi) = \frac{1}{d^N}\sum_{P\in\mathcal{P}_{N-1}}\sum_{\tilde{P}\in\mathcal{P}_1}\left|\sum_{i=0}^{d-1}\sum_{j=0}^{d-1}\langle i|\tilde{P}|j\rangle\langle\phi_i|P|\phi_j\rangle\sqrt{p_ip_j}\right|^{2\alpha} \tag{25}$$

$$= \frac{1}{d^N}\sum_{P\in\mathcal{P}_{N-1}}\sum_{a,b=0}^{d-1}\left|\sum_{i=0}^{d-1}\sum_{j=0}^{d-1}\langle i|X^aZ^b|j\rangle\langle\phi_i|P|\phi_j\rangle\sqrt{p_ip_j}\right|^{2\alpha} \tag{26}$$

$$= \frac{1}{d^N}\sum_{P\in\mathcal{P}_{N-1}}\sum_{a,b=0}^{d-1}\left|\sum_{i=0}^{d-1}\sum_{j=0}^{d-1}\langle i|j\oplus a\rangle\omega^{bj}\langle\phi_i|P|\phi_j\rangle\sqrt{p_ip_j}\right|^{2\alpha} \tag{27}$$

$$= \frac{1}{d^N}\sum_{P\in\mathcal{P}_{N-1}}\sum_{a,b=0}^{d-1}\left|\sum_{j=0}^{d-1}\omega^{bj}\langle\phi_i|P|\phi_j\rangle\sqrt{p_{j\oplus a}p_j}\right|^{2\alpha} \tag{28}$$

$$= \frac{1}{d^N}\sum_{P\in\mathcal{P}_{N-1}}\sum_{a,b=0}^{d-1}\sum_{|\boldsymbol{i}|=\alpha,|\boldsymbol{j}|=\alpha}\binom{\alpha}{\boldsymbol{i}}\binom{\alpha}{\boldsymbol{j}}\left(\prod_{m=0}^{d-1}\omega^{bm(j_m-i_m)}\right)\left(\prod_{m=0}^{d-1}\langle\phi_{m\oplus a}|P|\phi_m\rangle^{j_m}\langle\phi_m|P^\dagger|\phi_{m\oplus a}\rangle^{i_m}\right)\left(\prod_{m=0}^{d-1}(p_{j\oplus a}p_j)^{j_m+i_m}\right),$$

where in the second step we substituted $\tilde{P} = X^aZ^b$ for $a,b = 0,\dots,d-1$, and in the last step we introduced the multinomial indices $\boldsymbol{i} = (i_0,\dots,i_{d-1})$ with $0\leq i_m\leq 1$ and $|\boldsymbol{i}|\equiv\sum_{m=0}^{d-1}j_m$, while

$$\binom{\alpha}{\boldsymbol{i}} = \frac{\alpha!}{\prod_{m=0}^{d-1}i_m!}\,. \tag{29}$$

Summing over the b, we have a delta function forcing $\sum_{m=0}^{d-1}m(j_m-i_m) = 0 \mod d$. We denote this condition $\tilde{\delta}_{\boldsymbol{i},\boldsymbol{j}}\equiv\delta_{\sum_m m(j_m-i_m)=0 \mod d}$ for convenience. At the same time, we define $f^{(a)}_{\boldsymbol{i},\boldsymbol{j}}(\boldsymbol{p})\equiv\prod_{m=0}^{d-1}(p_{j\oplus a}p_j)^{j_m+i_m}$ for later convenience. Collecting these results, we have

$$P_\alpha(\Psi) = \frac{1}{d^{N-1}}\sum_{P\in\mathcal{P}_{N-1}}\sum_{a=0}^{d-1}\sum_{|\boldsymbol{i}|=\alpha}\sum_{|\boldsymbol{j}|=\alpha}\binom{\alpha}{\boldsymbol{i}}\binom{\alpha}{\boldsymbol{j}}\tilde{\delta}_{\boldsymbol{i},\boldsymbol{j}}\left(\prod_{m=0}^{d-1}\langle\phi_{m\oplus a}|P|\phi_m\rangle^{j_m}\langle\phi_m|P^\dagger|\phi_{m\oplus a}\rangle^{i_m}\right)f^{(a)}_{\boldsymbol{i},\boldsymbol{j}}(\boldsymbol{p})\,. \tag{30}$$

Until now all computations are exact. We now bound the expression taking the modulus. We have

$$\left|\sum_{P\in\mathcal{P}_{N-1}}\prod_{m=0}^{d-1}\langle\phi_{m\oplus a}|P|\phi_m\rangle^{j_m}\langle\phi_m|P^\dagger|\phi_{m\oplus a}\rangle^{i_m}\right|\leq\sum_{P\in\mathcal{P}_{N-1}}\prod_{m=0}^{d-1}\left|\langle\phi_{m\oplus a}|P|\phi_m\rangle^{j_m}\langle\phi_m|P^\dagger|\phi_{m\oplus a}\rangle^{i_m}\right| \tag{31}$$

$$\leq\sum_{P\in\mathcal{P}_{N-1}}\prod_{m=0}^{d-1}\left|\langle\phi_{m\oplus a}|P|\phi_m\rangle\right|^{j_m+i_m} \tag{32}$$

$$\leq\prod_{m=0}^{d-1}\left(\sum_{P\in\mathcal{P}_{N-1}}|\langle\phi_m|P|\phi_{m\oplus a}\rangle|^{2\alpha}\right)^{\frac{i_m+j_m}{2\alpha}}, \tag{33}$$

where in the last step we used Holder's inequality. Next, by manipulating the Pauli strings we get

$$\sum_{P\in\mathcal{P}_{N-1}}|\langle\phi_m|P|\phi_{m\oplus a}\rangle|^{2\alpha}\leq\left(\sum_{P\in\mathcal{P}_{N-1}}|\langle\phi_m|P|\phi_m\rangle|^{2\alpha}\right)^{1/2}\left(\sum_{P\in\mathcal{P}_{N-1}}|\langle\phi_{m\oplus a}|P|\phi_{m\oplus a}\rangle|^{2\alpha}\right)^{1/2}. \tag{34}$$

This follows expanding the expression

$$\sum_{P\in\mathcal{P}_{N-1}} |\langle\phi_m|P|\phi_{m\oplus a}\rangle|^{2\alpha} = \sum_{P\in\mathcal{P}_{N-1}} \mathrm{tr}(P|\phi_m\rangle\langle\phi_m|P^\dagger|\phi_{m\oplus a}\rangle\langle\phi_{m\oplus a}|) \tag{35}$$

$$= \frac{1}{d^{\alpha(N-1)}}\sum_P \sum_{P_1,\dots,P_\alpha}\prod_{i=1}^{\alpha}\left(\mathrm{tr}(P_i|\phi_m\rangle\langle\phi_m|)\mathrm{tr}(P_i^\dagger|\phi_{m\oplus a}\rangle\langle\phi_{m\oplus a}|)\right)\prod_{i=1}^{\alpha}K(P,P_i)\ , \tag{36}$$

where $K(P,Q)=\mathrm{tr}(PQP^\dagger Q^\dagger)/d^{N-1}$. Since $K(P,Q_1)K(P,Q_2)=K(P,Q_1Q_2)$ we have

$$\sum_P\prod_{i=1}^{\alpha}K(P,P_i)=\sum_P K(P,P_1\dots P_\alpha)=d^{2(N-1)}\delta_{P_1\dots P_\alpha,\mathbb{I}}. \tag{37}$$

Thus

$$\sum_{P\in\mathcal{P}_{N-1}} |\langle\phi_m|P|\phi_{m\oplus a}\rangle|^{2\alpha} = \frac{1}{d^{(\alpha-2)(N-1)}}\sum_{P_1,\dots,P_\alpha}\delta_{P_1\dots P_\alpha,\mathbb{I}}\prod_{i=1}^{\alpha}\langle\phi_m|P_i|\phi_m\rangle\langle\phi_m|P_i^\dagger|\phi_m\rangle\ , \tag{38}$$

obtaining Eq. (34) after using Cauchy-Schwarz for the $d^{2\alpha(N-1)}$ dimensional vectors with components

$$\delta_{P_1\dots P_\alpha,\mathbb{I}}\mathrm{tr}(P_1|\phi_k\rangle\langle\phi_k|)\dots\mathrm{tr}(P_\alpha|\phi_k\rangle\langle\phi_k|)\ . \tag{39}$$

From Eq. (33), multiplying and dividing by factors d^{N-1}, recognizing the definition of P_α and collecting these statements we obtain

$$P_\alpha(\Psi)\le\sum_{a=0}^{d-1}\sum_{|\boldsymbol{i}|=\alpha}\sum_{|\boldsymbol{j}|=\alpha}\binom{\alpha}{\boldsymbol{i}}\binom{\alpha}{\boldsymbol{j}}\tilde{\delta}_{\boldsymbol{i},\boldsymbol{j}}\prod_{m=0}^{d-1}\left(P_\alpha(\phi_m)^{\frac{i_m+j_m}{4\alpha}}P_\alpha(\phi_{m\oplus a})^{\frac{i_m+j_m}{4\alpha}}\right)f_{\boldsymbol{i},\boldsymbol{j}}^{(a)}(\boldsymbol{p})\ . \tag{40}$$

Denoting $\max_m\{P_\alpha(\phi_m)\}$ the maximal value of P_α over the $|\phi_m\rangle$ states, we have

$$P_\alpha(\Psi)\le\max_m\{P_\alpha(\phi_m)\}\sum_{|\boldsymbol{i}|=\alpha}\sum_{|\boldsymbol{j}|=\alpha}\binom{\alpha}{\boldsymbol{i}}\binom{\alpha}{\boldsymbol{j}}\tilde{\delta}_{\boldsymbol{i},\boldsymbol{j}}\sum_{a=0}^{d-1}f_{\boldsymbol{i},\boldsymbol{j}}^{(a)}(\boldsymbol{p})\ . \tag{41}$$

We note that

$$\sum_{a=0}^{d-1}f_{\boldsymbol{i},\boldsymbol{j}}^{(a)}(\boldsymbol{p})\le d^{1-2\alpha}\ , \tag{42}$$

and furthermore

$$\sum_{|\boldsymbol{i}|=\alpha}\sum_{|\boldsymbol{j}|=\alpha}\binom{\alpha}{\boldsymbol{i}}\binom{\alpha}{\boldsymbol{j}}\tilde{\delta}_{\boldsymbol{i},\boldsymbol{j}}d^{1-2\alpha}\le 1. \tag{43}$$

Summarizing these statements, we have

$$P_\alpha(\Psi)\le\max_m\{P_\alpha(\phi_m)\}\sum_{|\boldsymbol{i}|=\alpha}\sum_{|\boldsymbol{j}|=\alpha}\binom{\alpha}{\boldsymbol{i}}\binom{\alpha}{\boldsymbol{j}}\tilde{\delta}_{\boldsymbol{i},\boldsymbol{j}}\sum_{a=0}^{d-1}f_{\boldsymbol{i},\boldsymbol{j}}^{(a)}(\boldsymbol{p})\le\max_m\{P_\alpha(\phi_m)\} \tag{44}$$

concluding the proof of the Lemma. The monotonicity of the stabilizer Rényi entropy follows as a corollary. □

B Generalized Stabilizer entropies are not monotones

To study the monotonicity of GSE under computational basis measurements, we consider (without loss of generality) a measurement of the first qutrit and expand the pure state $|\Psi\rangle$ of N qudits as

$$|\Psi\rangle=\sum_{i=0}^{d-1}\sqrt{p_i}|i\rangle\otimes|\phi_i\rangle, \tag{45}$$

where $\{|i\rangle\}_{i=0}^{d-1}$ are the computational basis states, $\sum_{i=0}^{d-1}p_i=1$, and $|\phi_i\rangle$ are the states of the remaining $N-1$ qudits.

In the following, by analyzing the behavior of M_Y under measurements in the computational basis, we demonstrate that this GSE is not a monotone. To that end we find an example of a $N = 2$ qutrits state for which (23) is not satisfied and M_Y increases when a measurement in the computational basis is performed. We consider a state $|\Psi_2\rangle = \sum_{i,j=0}^{2} a_{i,j}|i\rangle \otimes |j\rangle$, where $a_{00} = 0.04899 + 0.29503i$, $a_{10} = 0.11566 + 0.04942i$, $a_{20} = 0.24715 + 0.34531i$, $a_{01} = 0.37355 + 0.23511i$, $a_{11} = 0.15912 + 0.33928i$, $a_{21} = 0.33144 + 0.08953i$, $a_{02} = 0.10273 + 0.08267i$, $a_{12} = 0.43260 + 0.22725i$, $a_{22} = 0.02948 + 0.06498i$). For this state, we have $Y_3(|\Psi_2\rangle\langle\Psi_2|) \approx 0.7837$, which is smaller than the GSE values for each of the states obtained after the measurement of the first qutrits in computational basis: $Y_3(|\phi_1\rangle\langle\phi_1|) \approx 1.0004$, $Y_3(|\phi_2\rangle\langle\phi_2|) \approx 1.0408$, $Y_3(|\phi_3\rangle\langle\phi_3|) \approx 1.4456$. This counter-example shows that not all nonstabilizerness measures induced by the defect subspaces of the Clifford commutant are monotones.

SUPPLEMENTARY NOTE 4: TENSOR NETWORK COMPUTABILITY

In this section, we argue that GSEs can be efficiently computed for any defect subspace $\mathcal{A}$ using tensor network protocols. A matrix product state (MPS) $|\Psi_N\rangle$ is defined by

$$|\Psi_N\rangle = \sum_{\{s_k \in \mathbb{Z}_d\}_k} A_{[1]}^{s_1} A_{[2]}^{s_2} \cdots A_{[N]}^{s_N} |s_1, \ldots, s_N\rangle, \tag{46}$$

where $A_i^{s_i}$ are $\chi_i \times \chi_{i+1}$ matrices for any $i = 2, \ldots, N-1$, and $A_1^{s_1}$ ($A_N^{s_N}$) are $1 \times \chi_2$ ($\chi_N \times 1$) matrices. Here, in contrast to the Main Text, we are considering the state of N qudits. For simplicity, we assume $\chi = \chi_i$ for any $i = 1, \ldots, N$, and refer to χ as the bond dimension. The following ideas extend straightforwardly to translationally-invariant states. As discussed in the Main Text, $\zeta_W = \mathrm{tr}(w^{\otimes N} |\Psi\rangle\langle\Psi|^{\otimes k})$. Given that $w \geq 0$, it can be decomposed via $w = \Gamma^\dagger \Gamma$. This decomposition allows us to define a new tensor

$$B_{[i]}^{\tilde{s}_i} = \Gamma \cdot A_i^{\otimes k}, \tag{47}$$

with a bond dimension χ^k and a physical dimension $\mathrm{rank}(w)$. The corresponding replica MPS is

$$|\Phi^{(N)}\rangle \equiv \sum_{\{\tilde{s}_k \in \mathcal{A}\}_k} B_{[1]}^{\tilde{s}_1} B_{[2]}^{\tilde{s}_2} \cdots B_{[N]}^{\tilde{s}_N} |\tilde{s}_1, \ldots, \tilde{s}_N\rangle, \tag{48}$$

and the computation of magic resources simplifies to computing the norm

$$M_W(|\Psi_N\rangle) = -\log\langle\Phi^{(N)}|\Phi^{(N)}\rangle. \tag{49}$$

In a similar fashion, the MPS formulation facilitates efficient sampling methods, such as Pauli Monte-Carlo and perfect sampling, as well as compression algorithms for tensor networks.

SUPPLEMENTARY NOTE 5: GENERALIZED STABILIZER ENTROPY OF HAAR RANDOM STATES

We discuss the GSEs of Haar random states. Due to the concentration of the Haar measure on the unitary group for $N \gg 1$, we have $\overline{M_W} \equiv \mathbb{E}_{\mathrm{Haar}}[\zeta_W] = -\log[\mathbb{E}_{\mathrm{Haar}}[\zeta_W]] + O(\exp(-\gamma N))$. Recall from Ref. [1] that:

$$\mathbb{E}_{\mathrm{Haar}}[|\Psi\rangle\langle\Psi|^{\otimes k}] = \frac{(d^N - 1)!}{(d^N + k - 1)!} \sum_{\pi \in S_k} W(T_\pi), \tag{50}$$

where T_π is the stochastic Lagrangian subspace induced by the permutation $\pi \in S_k \subset \mathcal{O}_k$. Thus, we find for the $W = W(T)$ of interest

$$\overline{\zeta_{W(T)}} = \mathbb{E}_{\mathrm{Haar}}[\zeta_{W(T)}] = \frac{(d^N - 1)!}{(d^N + k - 1)!} \sum_{\pi \in S_k} [\mathrm{tr}(w(T) w(T_\pi))]^N = \frac{(d^N - 1)!}{(d^N + k - 1)!} \sum_{\pi \in S_k} d^{N|T \cap T_\pi|}, \tag{51}$$

where $|T \cap T_\pi|$ is the cardinality of the intersection between the stochastic lagrangian subspaces. We specialize to the case of M_2 for qubits and qutrits with $k = 4$ replicas, and M_Y for qutrits with $k = 3$ replicas. In these cases we have

$$\mathrm{tr}(w(T) w(T_\pi)) = \begin{cases} d^{D-1} & \pi = (), \\ d^2 & \pi \in \{(12\ldots D), (23\ldots D1), \ldots, (D1\ldots 2)\}, \\ d^3 & \pi \in S_2 \otimes S_2, \text{ and } d = 2, \\ d^3 & \pi \in \{(13)(24), (14)(23)\} \text{ and } d = 3, \\ d & \pi = (12)(34) \text{ and } d = 3, \\ d^{\#(\pi)-1} & \text{otherwise,} \end{cases} \tag{52}$$

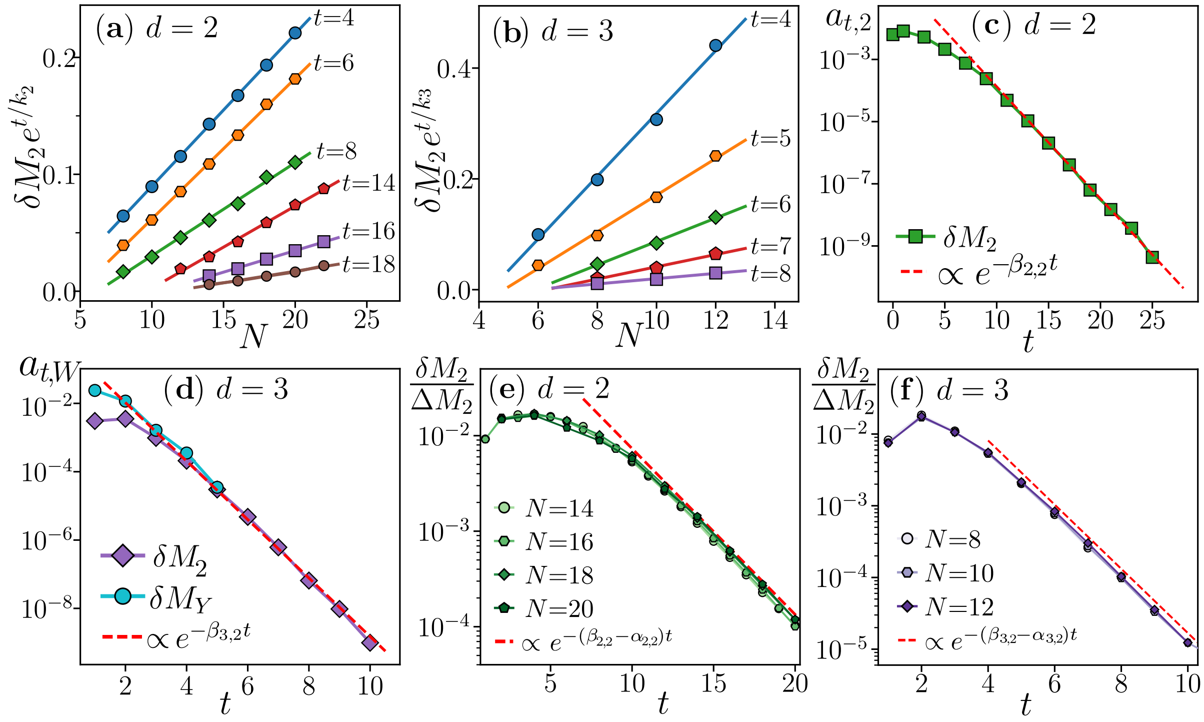

Supplementary Figure 1. Self-averaging of SRE and GSE. For qubits, (**a**), and qutrits, (**b**), the difference δM_2 between the annealed and quenched averages of SRE scales linearly with the system size N, c.f., (53) (for presentation purposes δM_2 is rescaled by a factor e^{t/k_d} with $k_2 = 3$ and $k_3 = 5/7$). The coefficient a_t, shown in (**c**) and (**d**), describing the leading term in this dependence, decays exponentially in time t, with characteristic rates $\beta_{2,2} = 0.83(3)$ and $\beta_{3,2} = 1.97(5)$. The relative error decays exponentially in time, (55), see (**e**) and (**f**).

where $\#(\pi)$ is the number of cycles. Combining these results, we recast M_W^{Haar} for the GSEs of interest in the Main Text.

SUPPLEMENTARY NOTE 6: ADDITIONAL NUMERICAL RESULTS

In this section, we provide additional numerical details which underlie the methodology of this work and expand the scope of our results.

A Self-averaging

In this section we detail the self-averaging analysis presented in Methods. We employ the exact numerical simulation to calculate the quenched average of the GSEs $\overline{M}_W = -\mathbb{E}[\ln[\zeta_W(|\Psi_t\rangle)]]$ and the annealed average $\tilde{M}_W = -\ln[\mathbb{E}[\zeta_W(|\Psi_t\rangle)]]$, where the circuit average involves 1000 realizations unless otherwise specified. The results are summarized in Supplementary Fig. 1.

As shown in Supplementary Fig. 1.(a), the difference $\delta M_W(t) = |\overline{M}_W(t) - \tilde{M}_W(t)|$ between the quenched and annealed averages exhibits a linear system size dependence give by

$$\delta M_2(t) = a_{t,2}N + b_t, \tag{53}$$

where $a_{t,2}$ and $b_{t,2}$ are constant at fixed time t. This holds for both qubits ($d = 2$) and qutrits ($d = 3$).

In Supplementary Fig. 1(c),(d), we show that at large time, the coefficients $a_{t,W}$ decreases exponentially over time for all relevant operators W in both qubit and qutrit systems

$$a_{t,W} = a_W e^{-\beta_{d,W}t}, \tag{54}$$

where $\beta_{d,W}$ is a constant dependent on the on-site Hilbert space dimension d and on W. We have presented these data in the Methods section: for qubits, we find $\beta_{2,2} = 0.83(3)$ while for qutrits $\beta_{3,2} = \beta_{3,Y} = 1.97(5)$. In that section, we

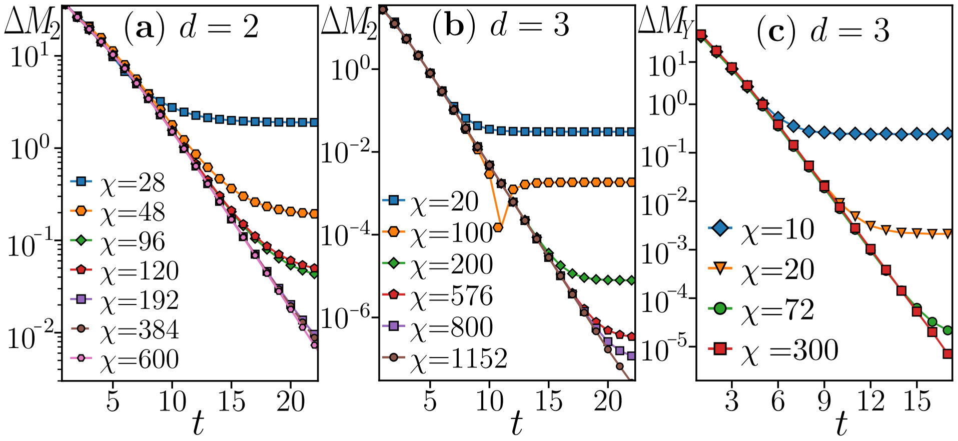

Supplementary Figure 2. Convergence of the tensor network contraction for $\Delta M_2(t)$ and $\Delta M_Y(t)$ with bond dimension χ and systems comprising $N = 64$ qubits (**a**) and qutrits (**b**, **c**). Increase of the bond dimension around $\chi = O(q_{\rm eff}^2)$ leads to no significant changes in the value of $\Delta M_W = M_W^{\rm Haar} - \tilde{M}_W(t)$, indicating the convergence of the results.

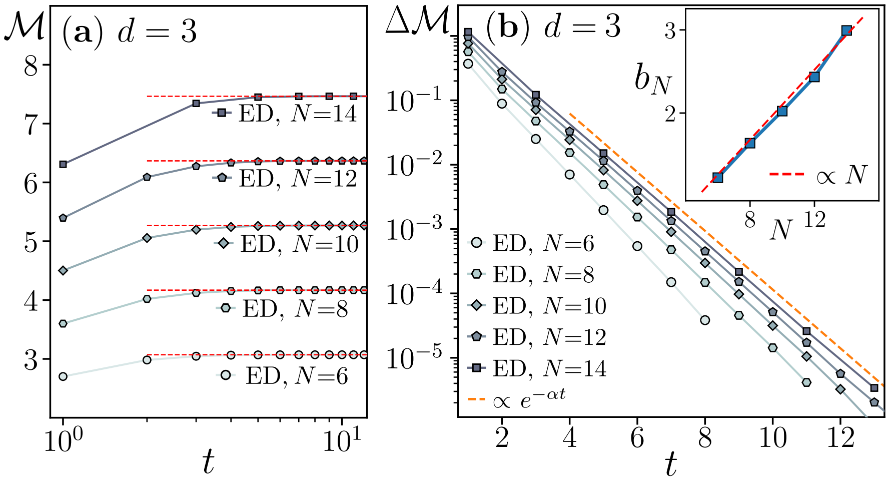

Supplementary Figure 3. Dynamics of mana $\mathcal{M}$ for a system of N qutrits ($d = 3$) under Haar random circuits. (**a**) Saturation of $\mathcal{M}$ to $\mathcal{M}^{\rm Haar}$ occurs rapidly with the circuit depth t. (**b**) The difference $\Delta\mathcal{M} = \mathcal{M}^{\rm Haar} - \mathcal{M}$ follows $\Delta M_2 \propto Ne^{-\alpha t}$ with $\alpha_3 = 1.05(9)$ at $t \gtrsim 4$. The annealed averages $\mathcal{M}$ over more than 100 circuit realizations are calculated with the exact numerics (ED) for all N.

commented that these results confirms that the quenched-to-annealed approximation error is negligible for sufficiently large N and t. As presented in Supplementary Fig. 1(e),(f), in fact, the relative error

$$\delta M_W(t)/\Delta M_W(t) \propto e^{-(\beta_{d,W}-\alpha_{d,W})t}, \tag{55}$$

decay exponentially in time up to a sub-leading in system size term $O(1/N)$. In this figure, we observe a clear exponential decay of the relative error starting at times $\approx 5-10$ at which the saturation of GSEs is observed for the system sizes considered in this work.

B Tensor network contractions

In this section, we detail the bond dimension required for the saturation of our data.

Specifically, in Supplementary Fig. 2, we show the dependence of the tensor network contraction results for $\Delta\tilde{M}_W(t)$ for both qubits and qutrits as a function of the bond-dimension χ. The results cease to be χ-dependent once χ is of order of $q_{\rm eff}^2$. Specifically, computation of the SRE $\Delta\tilde{M}_2(t)$ for qubits is well-converged with χ already at $\chi = 192$, see Supplementary Fig. 2(a). In contrast, convergence of the $\Delta\tilde{M}_2(t)$ calculation for times $t \in [0, 18]$ for qutrits occurs

only at $\chi = 800$, as shown in Supplementary Fig. 2(b). This demonstrates how the reduction $q_{\text{eff}} = 24 \to q'_{\text{eff}} = 14$, unavailable for qutrits, diminishes the computational resources in the calculations for qubits. (Using irreducible representations reduces q_{eff} to 23 for qutrits, maintaining similar limitations as the full 24-dimensional case.). Finally, Supplementary Fig. 2(c) presents the calculations of $\Delta M_Y(t)$ for qutrits which require significantly smaller resources and are converged for $t \in [0, 15]$ already for $\chi = 300$. By varying the system size, we have verified that the conclusions for the convergence with bond dimension are nearly independent of system size as long as $N \gtrapprox 30$. The results presented in the Main Text are converged with the bond dimension χ.

C Results beyond generalized stabilizer entropies: mana

Mana offers insights into magic state resources as it is a strong nonstabilizerness monotone both for pure and mixed states. In the following, we resort to exact numerical computation of the time evolution of mana under brick-wall Haar random circuits for $N \le 14$. Our results are shown in Supplementary Fig. 3. We observe that mana becomes extensive, $\mathcal{M} \propto N$ already at $t = 1$, after a single layer of the circuit. Subsequently, $\mathcal{M}$ quickly saturates with t to the value $\mathcal{M}^{\text{Haar}}$ of the Haar-random state of N qutrits. As shown in Supplementary Fig. 3 (b), the difference $\Delta\mathcal{M} = \mathcal{M}^{\text{Haar}} - \mathcal{M}(t)$ exhibits a clear exponential decay, $\Delta\mathcal{M} = b_n e^{-\alpha t}$, with circuit depth t. The prefactor b_n increases linearly with N, as shown in the inset in Supplementary Fig. 3 (b). Moreover, the numerical results suggest that the value of α is converging with the increase of N towards a constant value. While the available range of N is not sufficient for a clear demonstration that the saturation time of mana follows $t_{\text{sat}}^{(\mathcal{M})} \propto \ln N$, the behavior of the coefficients α and b_N strongly suggests that $\Delta\mathcal{M} \propto N e^{-\alpha t}$, analogously to the GSEs case. This demonstrates the universality of the uncovered phenomenology of magic spreading among different measures of nonstabilizerness.

D Results beyond generalized stabilizer entropies: Stabilizer Rényi entropy growth under doped Clifford circuits

In the Main Text, we argued that the brick-wall random Haar circuits serve as minimal models of generic many-body dynamics. In particular, each layer of the Haar circuit contains extensively many 2-body gates, each generating a non-vanishing amount of the GSE.

There are, however, instances in which the resources generating non-stabilizerness are much more sparse which hinders the growth of the quantum magic resources. To illustrate this point, we consider an example of a doped Clifford circuit, in which 2-qubit unitary gates $U_{i,i+1}^{(C)}$ that form a brick-wall lattice are drawn with uniform probability from the Clifford group $\mathcal{C}_{2,2}$. The consecutive layers of the circuit are interspersed with action of a randomly placed T-gate defined as $T|m\rangle = e^{-i\pi m/4}|m\rangle$, which is the sole non-stabilizerness generating ingredient of the circuit's dynamics.

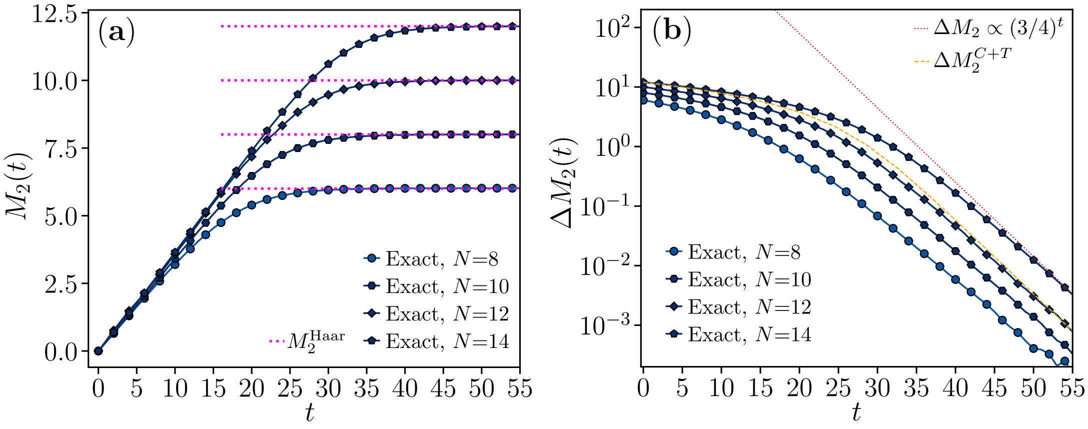

Supplementary Figure 4. Doped Clifford circuit simulations. (a) The evolution of $Y_2(t)$ obtained via exact numerics for system sizes $N \le 14$. The magenta dotted lines are the value at $t \to \infty$. (b) Crossover behavior of the difference $\Delta M_2(t) \equiv M_2^{\text{Haar}} - M_2(t)$ to a regime of exponential decay, $\Delta M_2 \propto (3/4)^t$ (denoted by the red dotted line). The formula ΔM_2^{C+T} (56) (denoted by the orange dashed line) qualitatively reproduces the bahavior of ΔM_2. The results are averaged over more than 1000 circuit realizations.

Our exact numerical results for the evolution of the SRE $M_2(t)$ obtained for this setup are summarized in Sup-

plementary Fig. 4. At small times, the SRE increases linearly with time, reflecting the gradual generation of magic resources by the T-gate and their spreading due to the Clifford dynamics. At long times, the SRE saturates to value M_2^{Haar}, and the approach to the saturation value, captured by $\Delta Y_2(t) = M_2^{\mathrm{Haar}} - M_2(t)$, becomes exponential in time, $\Delta M_2(t) \propto e^{-\alpha_C t}$, where $\alpha_C > 0$ is a constant. Incidentally, we observe that our numerical results for doped Clifford circuits are qualitatively reproduced by the formula

$$\Delta M_2^{C+T}(t) = M_2^{\mathrm{Haar}} + \log[M_2^{\mathrm{Haar}} + (3/4)^t], \tag{56}$$

obtained in [4, 5] for circuits in which each layer of the Clifford gates is replaced by a global, N-qubit, Clifford gate. While Eq. (56) corresponds to a circuit in which the non-stabilizerness spreading is more efficient, the linear growth of $M_2(t)$ at small times, as well as, the crossover to a regime of exponential relaxation towards the saturation value M_2^{Haar} are reflected by this equation. The latter behavior resembles the phenomenology of the brick-wall Haar random quantum circuits considered in the Main Text. Notably, however, the saturation of the SRE up to a fixed tolerance occurs at $t_{\mathrm{sat}}^{(C+T)} \propto N$, i.e., at times much longer than $t_{\mathrm{sat}}^{\mathrm{mag}}$ scaling logarithmically with N for generic many-body dynamics.

The doped Clifford circuits are, however, fine-tuned, i.e., a generic small perturbation δU of the Clifford gate $U_{i,i+1}^{(C)}$ results in a gate $U_{i,i+1}^{(C)} + \delta U$ which is no longer Clifford. Such a perturbed system is expected to follow the phenomenology of Haar circuits discussed in the Main Text.

E Rényi index dependence

The stabilizer Rényi entropy

$$M_q(|\Psi\rangle) = \frac{1}{1-q} \log\left[\sum_{P\in\mathcal{P}_N} \frac{\langle\Psi|P|\Psi\rangle^{2q}}{d^N}\right], \tag{57}$$

investigated in the Main Text for $q = 2$ can be studied for arbitrary Rényi index $q > 0$, in particular, the limiting cases $q = 1$ and $q = \infty$ are determined by the limits $q \to 1$ and $q \to \infty$ of (57). Computing SRE numerically, we observe that for $0 < q \leq 2$, the phenomenology of the SRE growth remains quantitatively the same as for $q = 2$ reported in the Main Text. A particular example is shown for $q = 1$ in Supplementary Fig. 5(a) in which we observe the characteristic exponential decay of $\Delta M_1(t) = M_1^{\mathrm{Haar}} - M_1(t)$ with a prefactor scaling linearly with the system size, and with saturation towards the Haar value occurring at times scaling logarithmically with the system size N. In contrast, for $q > 2$, see Supplementary Fig. 5(b),(c) for examples of $q = 3, 4$, we observe that the decay of ΔM_q to a small value $\epsilon \ll 1$ occurs at times that are independent of the system size N. This is consistent with observation of [6] that SRE of increasing index $q > 2$ progressively fail to distinguish highly magical Haar-random states from product states with limited nonstabilizerness. Nevertheless, the saturation of M_3 and M_4 is consistent with the results reported in the Main Text in the sense that M_2 (and $M_{0<q<2}$), M_Y, and $\mathcal{M}$ saturate at the longest possible time scale $t_{\mathrm{sat}}^{\mathrm{mag}} \propto \ln(N)$ at which the higher order SRE, $M_{q>2}$, are already saturated and remain agnostic to the changes of nonstabilizerness of the state.

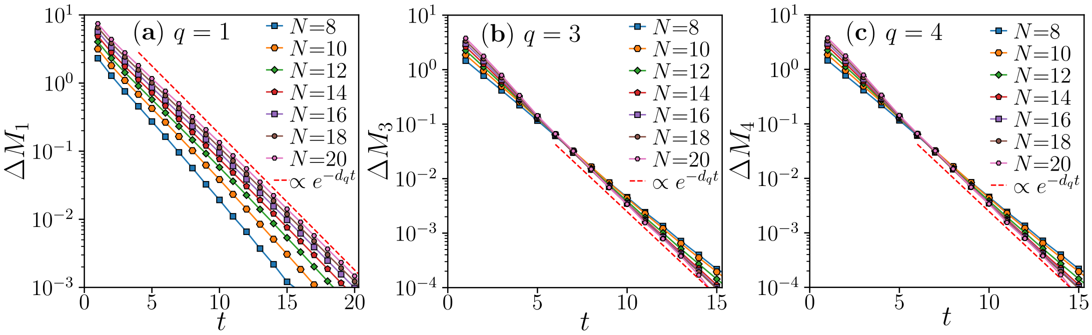

Supplementary Figure 5. Dynamics under Haar-random brick-wall circuit of SRE with Rényi indices $q = 1, 3, 4$ for systems of N qubits is shown in (a), (b), (c), respectively. The phenomenology of GSE from the Main Text is observed for $q = 1$. For $q = 3, 4$, the SRE saturates, up to a fixed accuraccy ϵ to its long-time value, at times which are independent of N.

[1] Gross, D., Nezami, S. & Walter, M. Schur–weyl duality for the clifford group with applications: Property testing, a robust hudson theorem, and de finetti representations. *Commun. Math. Phys.* **385**, 1325–1393 (2021).
[2] Montealegre-Mora, F. & Gross, D. Duality theory for clifford tensor powers (2024). URL https://arxiv.org/abs/2208.01688. arXiv: 2208.01688.
[3] Leone, L. & Bittel, L. Stabilizer entropies are monotones for magic-state resource theory. *Phys. Rev. A* **110**, L040403 (2024).
[4] Leone, L., Oliviero, S. F. E. & Hamma, A. Stabilizer rényi entropy. *Phys. Rev. Lett.* **128**, 050402 (2022).
[5] Haug, T., Aolita, L. & Kim, M. S. Probing quantum complexity via universal saturation of stabilizer entropies (2024). URL https://arxiv.org/abs/2406.04190. arXiv: 2406.04190.
[6] Turkeshi, X., Dymarsky, A. & Sierant, P. Pauli spectrum and nonstabilizerness of typical quantum many-body states. *Phys. Rev. B* **111**, 054301 (2025).
